# Microevolution in response to transient heme-iron restriction enhances intracellular bacterial community development and persistence

**DOI:** 10.1371/journal.ppat.1007355

**Published:** 2018-10-17

**Authors:** Rachael L. Hardison, Alistair Harrison, Rachel M. Wallace, Derek R. Heimlich, Meghan E. O’Bryan, Robert P. Sebra, Heather W. Pinkett, Sheryl S. Justice, Kevin M. Mason

**Affiliations:** 1 The Research Institute at Nationwide Children’s Hospital, Center for Microbial Pathogenesis, Columbus, Ohio, United States of America; 2 The Ohio State University College of Medicine, Infectious Diseases Institute, Columbus, Ohio, United States of America; 3 Icahn Institute and Dept. of Genetics & Genomic Sciences, Icahn School of Medicine at Mount Sinai, New York, New York, United States of America; 4 Department of Molecular Biosciences, Weinberg College of Arts & Sciences Northwestern University, Chicago, Illinois, United States of America; University of Maryland, UNITED STATES

## Abstract

Bacterial pathogens must sense, respond and adapt to a myriad of dynamic microenvironmental stressors to survive. Adaptation is key for colonization and long-term ability to endure fluctuations in nutrient availability and inflammatory processes. We hypothesize that strains adapted to survive nutrient deprivation are more adept for colonization and establishment of chronic infection. In this study, we detected microevolution in response to transient nutrient limitation through mutation of *icc*. The mutation results in decreased 3',5'-cyclic adenosine monophosphate phosphodiesterase activity in nontypeable *Haemophilus influenzae* (NTHI). In a preclinical model of NTHI-induced otitis media (OM), we observed a significant decrease in the recovery of effusion from ears infected with the *icc* mutant strain. Clinically, resolution of OM coincides with the clearance of middle ear fluid. In contrast to this clinical paradigm, we observed that the *icc* mutant strain formed significantly more intracellular bacterial communities (IBCs) than the parental strain early during experimental OM. Although the number of IBCs formed by the parental strain was low at early stages of OM, we observed a significant increase at later stages that coincided with absence of recoverable effusion, suggesting the presence of a mucosal reservoir following resolution of clinical disease. These data provide the first insight into NTHI microevolution during nutritional limitation and provide the first demonstration of IBCs in a preclinical model of chronic OM.

## Author summary

Nontypeable *Haemophilus influenzae* (NTHI) inhabits diverse niches in the host. The ability to adapt to new microenvironments is consistent with the predominance of NTHI as a causative agent of otitis media (OM) in children. We evaluated the microevolution of NTHI associated with adaptation and persistence in response to nutrient limitation. We identified a naturally occurring mutation that enhances NTHI persistence and formation of intracellular bacterial communities (IBCs) in a pre-clinical model of OM. The presence of IBCs during OM provides the first opportunity to evaluate the role of intracellular populations in chronicity and quiescence as a new paradigm for recurrent OM. This model provides a new platform to identify novel therapeutics for this highly prevalent and costly infectious disease.

## Introduction

The host sequesters essential micronutrients (i.e. metals) as a mechanism to limit bacterial infection [1]. Bacteria that require exogenous nutrients for growth are subjected to stresses that necessitate adaptation for survival. Bacteria can adapt to these stresses through transcriptional, epigenetic and genetic mechanisms [1]. Identification of genes subject to microevolution provides insight into the nutritional and environmental stresses incurred during infection as well as new therapeutic targets to quell infection.

Nontypeable *Haemophilus influenzae* (NTHI) is a heme auxotroph and must obtain this essential nutrient from the environment [2]. The presence of heme-containing compounds is sufficient to support polymicrobial growth in the nasopharynx [3]. During ascent from the nasopharynx to the middle ear, NTHI experiences fluctuations in nutrient availability, particularly heme-iron. The lack of sufficient heme-iron in the middle ear is indicated by the increase in the number of iron-uptake genes in NTHI strains isolated from children with otitis media [(OM); [4]]. Furthermore, iron-uptake genes are transcribed in NTHI early during experimental OM [5, 6]. We previously demonstrated that transient heme-iron restriction of NTHI potentiates changes in bacterial morphology, biofilm architecture, disease severity and provides a survival advantage in the preclinical model of OM [7].

NTHI inhabits diverse niches in the host including the nasopharynx, middle ear, sinus, eye and lung. At these sites, NTHI can be planktonic, in a biofilm or associated with mucosa [8]. NTHI has also been observed within intracellular niches *in vitro* in cultured lung and middle ear epithelial cells, as well as in clinical biopsies of adenoid and middle ear tissues from children with a history of OM [7, 9–12].

New paradigms for recurrent infections were revealed through the identification of intracellular bacterial communities (IBCs) in the cytoplasm of epithelial cells. This complex intracellular lifestyle, first described for uropathogenic *Escherichia coli* [(UPEC);[13]], provides access to nutrients and protection from antibiotic therapies and innate immune effectors [14, 15]. The IBC pathway is critical for acute urinary tract infection and culminates in a latent intracellular population that serves as a *bona fide* reservoir for recurrent infection [15]. Although first described in preclinical models, IBCs are present in bladder biopsies and in the urine of patients with urinary tract infections caused by UPEC [13]. IBCs of *Klebsiella* and *Proteus* have also been observed in the urinary tract [16, 17] and *Helicobacter pylori* in the stomach [18], in preclinical models of urinary tract infection and gastritis, respectively. We recently observed the formation of NTHI IBCs in cultured chinchilla middle ear epithelial cells in response to transient heme-iron limitation [7]. This observation suggests a potential role for intracellular populations of NTHI during OM and reveals new potential mechanisms for disease chronicity, persistence and recurrence that remain underexplored.

The ability to adapt to new microenvironments is consistent with the predominance of NTHI as a causative agent of OM, the most common bacterial infection in children [19–21]. Longitudinal sampling of patients with chronic obstructive pulmonary disease (COPD) revealed the same NTHI strains, despite intermittent periods of negative culture [22]. In this study, we evaluate the microevolution of NTHI associated with adaptation and persistence in response to nutrient limitation. In long-term stationary phase following transient heme-iron restriction, we observed a single nucleotide polymorphism in *icc* that abolishes 3’,5’-cyclic adenosine monophosphate (cAMP) phosphodiesterase activity. In the preclinical model for NTHI-induced OM, the *icc* mutant significantly shifts infection kinetics, with decreased middle ear fluid, a clinical indicator of disease resolution. However, the *icc* mutant was still associated with middle ear tissues at levels similar to the parental strain, suggesting that the presence of fluid and bacterial burden of the middle ear can be uncoupled. Although the bacterial burdens were similar, there was a significant increase in IBC formation by the *icc* mutant strain early during disease while formation of IBCs by the parental strain occurred gradually, indicating that NTHI adaptation to an intracellular niche occurs during active infection. Using UPEC as the paradigm, this observation suggests that NTHI intracellular populations may serve as a reservoir and contribute to both chronic and recurrent disease associated with OM.

## Results

### Transient heme-iron restriction promotes a cyclical pattern of viability and microevolution during stationary phase

**Fig 1:**
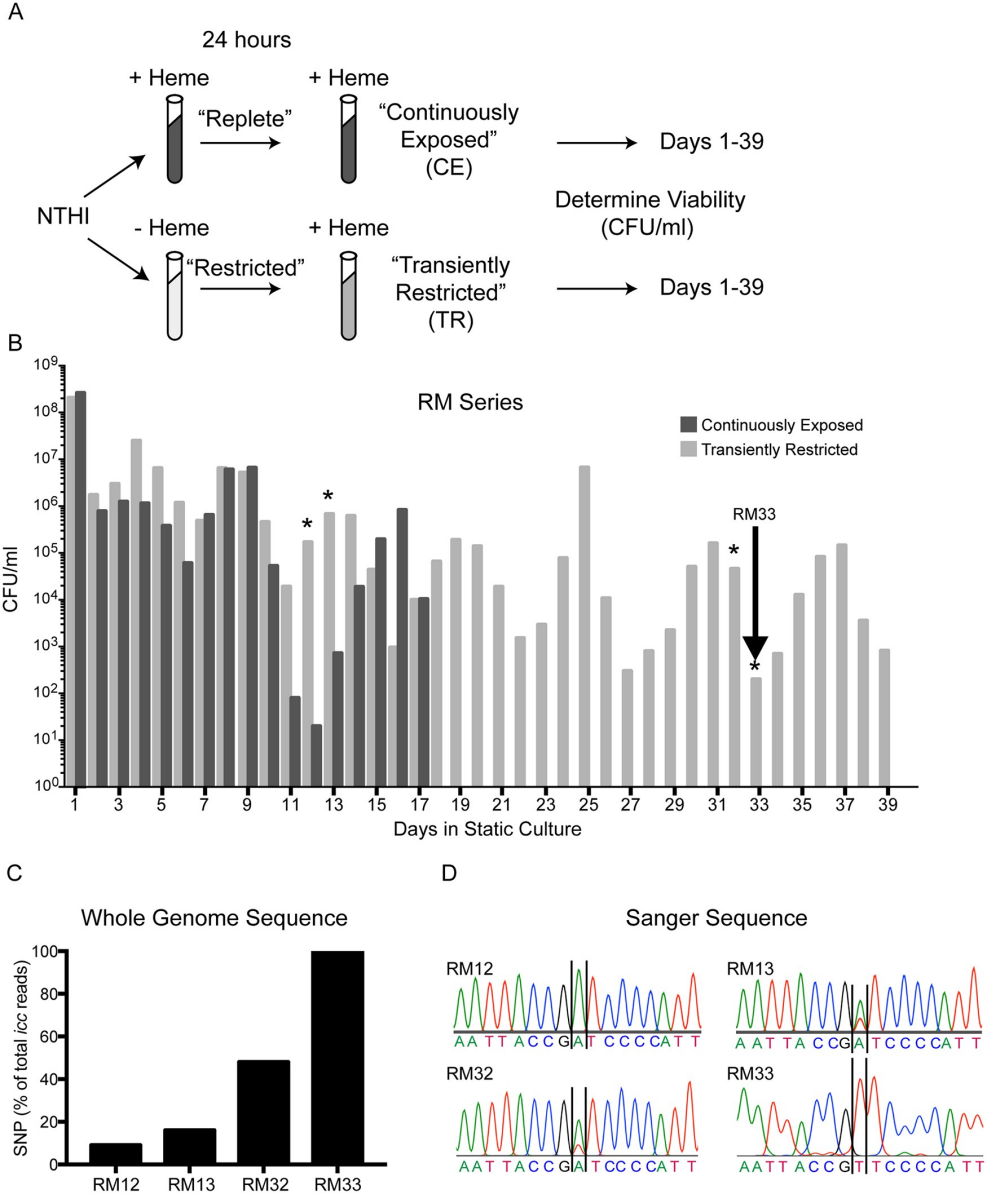
Transient heme-iron restriction promotes a cyclical pattern of viability and microevolution during stationary phase. (A) Schematic representation of environmental heme-iron restriction and extended incubation of stationary phase cultures. (B) Viability was determined every 24 hours for 39 days. Arrow indicates the culture selected for further investigation, termed RM33. Asterisks denote cultures selected for whole genome sequencing. (C) The sequence reads that exhibit the single nucleotide polymorphism (SNP) are reported as percentage of the total reads for *icc* on each of the days. The number of sequence reads for each sample were: RM12 (889), RM13 (632), RM32 (938) and RM33 (997). (D) Sanger sequencing electropherograms validate the mutation [Adenine (A) → Thymine (T)].

Bacterial subpopulations that arise stochastically contribute to persistence during infection. Formation of these populations can be enhanced by antibiotic exposure, oxidative stress, reduced indole signaling and nutrient limitation [as reviewed in [23]]. Based upon previous observations, we hypothesized that transient heme-iron restriction would invoke survival advantages to NTHI [7]. As a first approach to elucidate the physiological changes that contribute to persistence following transient restriction of heme-iron, we evaluated the longevity of NTHI in stationary phase. The prototypical NTHI strain 86-028NP was cultured in Defined Iron Source (DIS) medium in the presence or absence of 2 μg/mL heme-iron for 24 hours, then subcultured into DIS containing 2 μg/mL heme-iron, resulting in two parallel cultures, one continuously exposed to heme-iron and one transiently restricted of heme-iron (Fig 1A). The continuously exposed and transiently restricted NTHI cultures were incubated statically at 37°C, without the addition of fresh medium, and viability was assessed each day. The colonies obtained each day were pooled and cryopreserved to create a series of isolates (termed the “RM Series”) for further investigations. The NTHI cultures that were transiently restricted of heme-iron were viable for the entire duration of the experiment until the culture media was depleted (39 days) and exhibited repeated cycles of increasing and decreasing viability with a 6–7 day periodicity (Fig 1B). In marked contrast, the NTHI cultures continuously exposed to heme-iron were not viable beyond 17 days of incubation.

The cycling of growth during stationary phase can include the sequential acquisition and loss of single mutants defined as growth advantage in stationary phase [GASP; [24]]. To determine if mutations contributed to the observed phenotype, the whole genome sequence of RM33 isolated during the last cycle, day 33, was compared to the input strain, 86-028NP. Within the entire RM33 genome, only one single nucleotide variant was identified at position 63 of *icc*, which encodes a 3’,5’-cAMP phosphodiesterase. This mutation, an adenine to thymine nucleotide change, converts the 21^st^ amino acid in Icc from an aspartic acid to a valine. This base change was confirmed by Sanger sequencing (Fig 1D). To determine if other mutations were associated with the observed cycling, bacteria isolated on days 12, 13 and 32 (RM12, RM13 and RM32, respectively) were also subjected to whole genome sequencing. No additional mutations were identified at these earlier time points. We further observed that the sequences were derived from a mixed population of cells; the parental and the D21V mutation, with an enrichment for the *icc* mutant strain throughout the experiment (Fig 1C). The presence of the D21V mutation and the increase in mutant genotypes was also confirmed by Sanger sequencing (Fig 1D). On day 12, a single peak representing the parental adenine residue could be resolved. In contrast, in strains RM13 and RM32, although the adenine peak predominated, a smaller peak representing a thymine could be resolved. Finally, in strain RM33 only a peak representing a thymine could be resolved. No single nucleotide varients were detected in *icc* in the cultures that arose from the continuously exposed cultures (S1 Fig).

**Fig 2:**
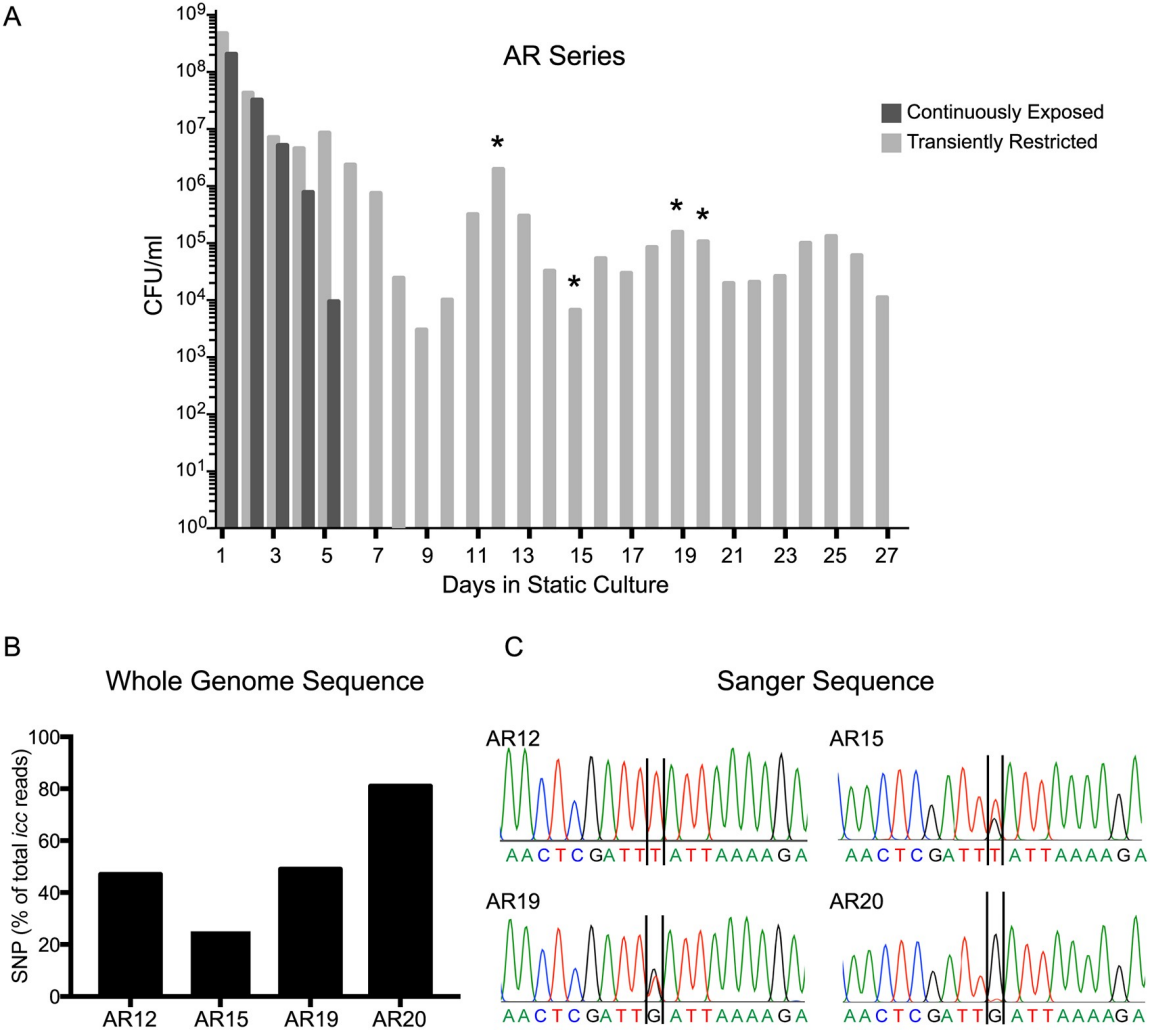
Independent validation of cyclical pattern of viability and microevolution. (A) Viability was determined every 24 hours for 27 days. Asterisks denote cultures selected for whole genome sequencing in the AR series. (B) The sequence reads that exhibit the SNP are reported as percentage of the total reads for *icc* on each of the days. The number of sequence reads for each sample were: AR12 (726), AR15 (707), AR19 (1021) and AR20 (865). (C) Sanger sequencing electropherograms validate the mutation [Thymine (T) → Guanine (G)].

A second independent experiment was performed to confirm that transient heme-iron restriction promotes long term survival during stationary phase. As with the previous study, viability of NTHI transiently restricted of heme-iron was observed for the duration of the experiment. In contrast, NTHI continuously exposed to heme-iron demonstrated reduced longevity in stationary phase (Fig 2A). In addition to the increased longevity, the 6–7 day periodicity of the cyclical increases and decreases in viability were also observed. To determine whether mutations also contributed to the phenotype in this series, whole genome sequencing was performed on the cultures from four different days. In this case, we observed three mutations that were confirmed by Sanger sequencing (Fig 2C). The first mutation resulted in a change in thymine to a guanine at position 434 in *icc*. This mutation generated a premature stop codon leading to a truncation at amino acid 144, resulting in a loss of the terminal 130 amino acids (Fig 2B). As observed with the RM series, the sequences from the AR series also showed mixed populations of the parental and the *icc* mutant with the proportion of the mutant increasing over time (Fig 2B and 2C). The second mutation was a deletion of one of the repeats in the phase variable region of *licA* and was observed in all samples analyzed. This change is consistent with phase variation observed for this gene [25]. The third mutation was a single nucleotide change that converts an alanine to a threonine at position 217 of *rne*, which encodes an endoribonuclease, and the frequency of mutation parallels that of the *icc* mutant. Although the mechanism of cycling viability is not understood, the absence of additional mutations within each cycle excludes the contribution of GASP, suggesting additional mechanisms of cyclical growth during stationary phase.

During exponential growth, no differences in viability of RM33 and 86-028NP were observed when grown under transiently restricted or continuously exposed conditions, either individually or in competitition (S2 and S3 Figs). When grown individually in long-term culture, continuously exposed RM33 survives 10 days longer than continuously exposed 86-028NP (S4A Fig). Further, RM33 outcompetes the parental strain in co-culture conditions (S4C and S4D Fig). In a direct competition between transiently restricted RM33 and continuously exposed RM33, prior restriction of heme-iron did not promote a further survival advantage, suggesting that RM33 is adapted to this nutritional limitation (S4B Fig). Interestingly, transient heme-iron restriction does provide some additional survival advantage to RM33 as indicated by an increase in the longevity by 5 days when in co-culture with the parental strain (S4C and S4D Fig). The presence of RM33 does not promote survival of the 86-028NP parental strain.

### Naturally occurring *icc* mutants demonstrate reduced cAMP phosphodiesterase activity

**Fig 3:**
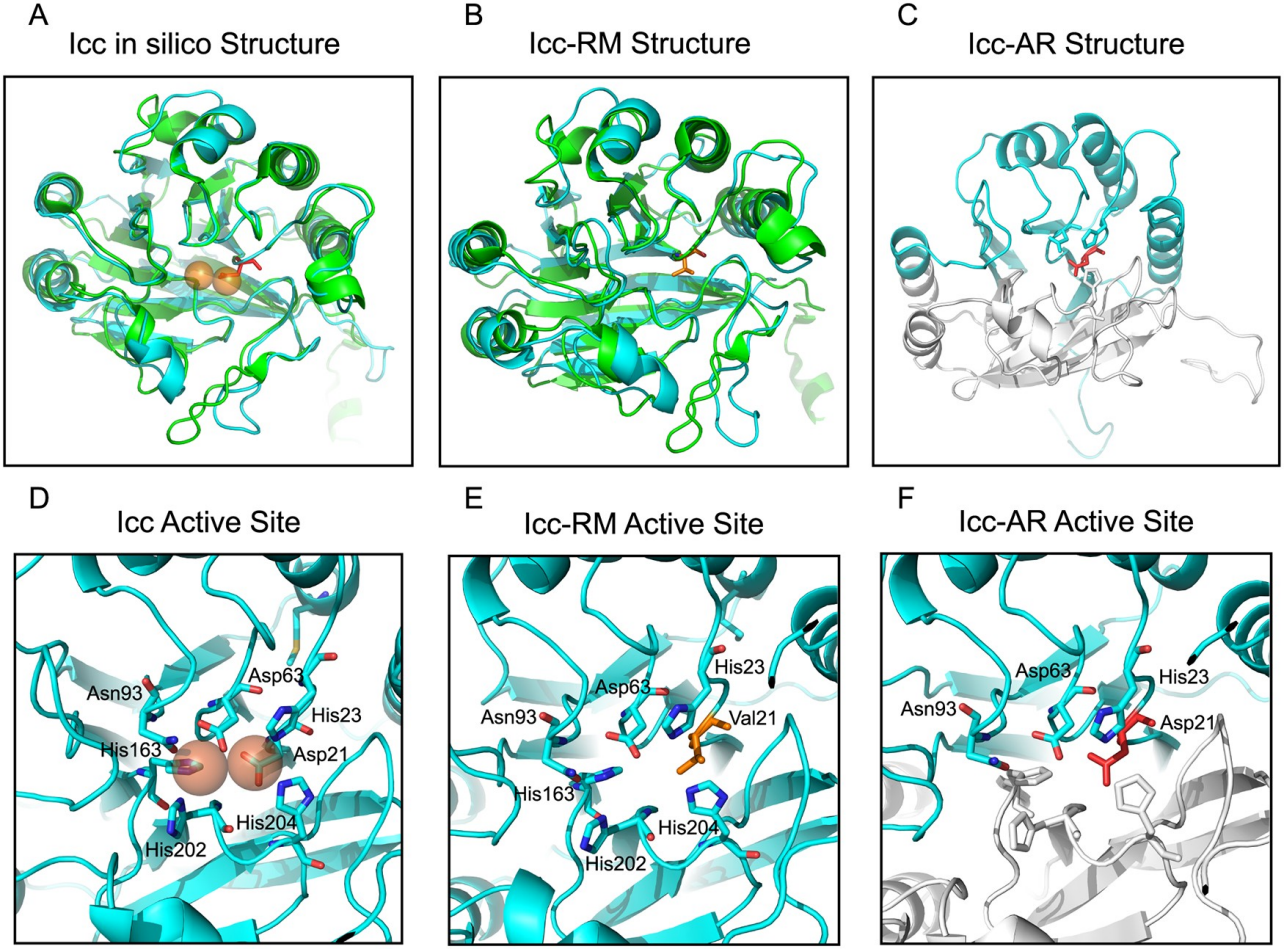
*In silico* structural modeling of Icc. (A) Computational model of NTHI Icc (colored cyan) aligned with the known three-dimensional structure of GpdQ [PDB ID:2ZO9, (colored green)] from *Enterobacter aerogenes*. The two orange spheres indicate the position of iron co-factors in the Icc active site and aspartic acid 21 is indicated in red. (B) The computational model of Icc-RM with the mutation at position 21 from an aspartic acid to a valine shown in stick formation (indicated in orange). (C) The computational model of NTHI Icc-AR (colored cyan) demonstrating the truncated protein was overlaid on the full length Icc (colored white). For reference amino acid 21 in the active site is shown in red. (D) Magnification of the NTHI Icc active site and iron co-factors (orange spheres) coordinated by the aspartic acid at position 21, histidine 23, aspartic acid 63, histidine 204, histidine 202, histidine 163 and asparagine 93. (E) Magnification of the Icc-RM active site with the aspartic acid to valine mutation depicted in orange. (F) Magnification of NTHI Icc-AR active site depicts the premature stop codon that disrupts the active site and iron coordination due to the lack of histidine 204, 202 and 163.

NTHI *icc* is an ortholog of *Escherichia coli cpdA*, which encodes a class III 3’, 5’-cAMP phosphodiesterase that degrades cyclic AMP [26]. Class III phosphodiesterases are characterized by a highly conserved active site with the sequence motif D-(X)_n_-GC-(X)_n_-GNH[E/D]-(X)_n_-GHXH [27]. This motif, which is also present in orthologs from NTHI and *Enterobacter aerogenes*, binds metal ions at the active site and is critical for the enzymatic function of class III phosphodiesterases [27]. The three-dimensional structure of the CpdA ortholog GpdQ from *E*. *aerogenes*, which has a perfect copy of the active site motif, has been solved [28]. To determine if NTHI Icc contained the conserved active site motif, SWISS-MODEL (https://swissmodel.expasy.org) was employed to construct a three-dimensional (3D) structure using crystal structures of homologs. The generated 3D model of NTHI Icc when aligned with the GpdQ structure reveals a similar overall topology (Fig 3A). The NTHI Icc model predicts a conservation of the active site where Asp21 contributes to metal coordination (Fig 3D). Mutations of this conserved residue, as observed in RM33, may result in a disruption of enzymatic activity [[27];Fig 3B and 3E]. The introduction of a premature stop codon at residue 145 in Icc in the AR series would result in a truncation of important active site residues that disrupt the two C-terminal metal-binding domains (Fig 3C and 3F). Thus, we hypothesize that both of the naturally occurring mutations in *icc* would affect cAMP phosphodiesterase activity.

**Fig 4:**
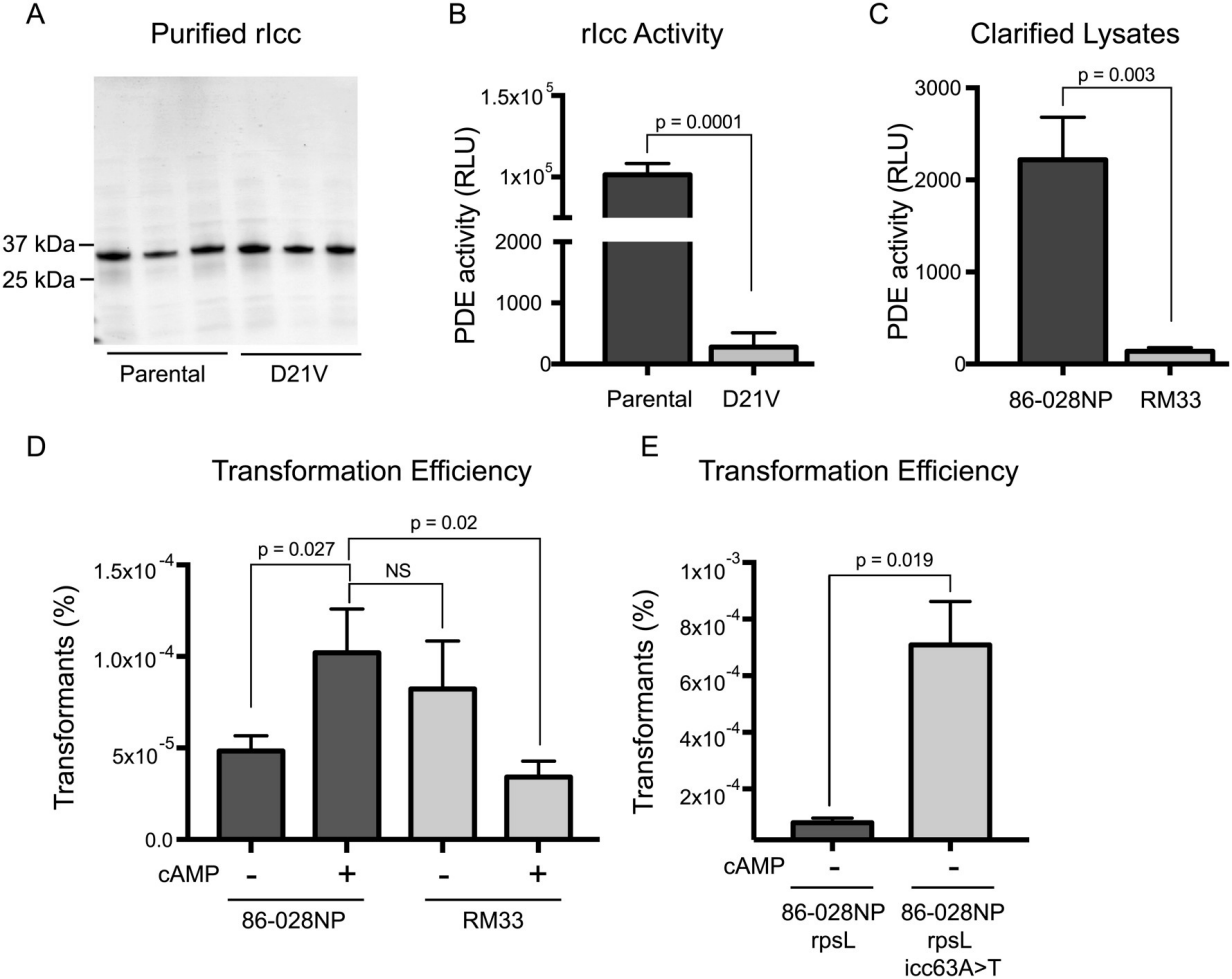
Biochemical and functional assays confirm reduced cAMP phosphodiesterase activity of mutant Icc. (A) Purified recombinant parental and mutant Icc proteins were visualized on a silver stained SDS polyacrylamide gel. Icc migrates with an apparent molecular weight of 32kDa. Each lane represents purification of the protein from an independent culture. (B) Phosphodiesterase activity (PDE) of purified recombinant proteins was assessed using the PDELight HTS cAMP phosphodiesterase kit and activity reported as relative light units (RLU). Three biological replicates were assayed in technical triplicate and statistical significance was determined using a one–tailed Student’s *t* test. The mean is plotted and error bars indicate standard error of the mean (SEM). (C) Bacterial lysates of the parent (86-028NP) and mutant (RM33) strains were assessed for phosphodiesterase activity as described in panel B. Four biological replicates were assayed in technical triplicate and statistical significance was determined using a one–tailed Student’s *t* test. The mean is plotted and error bars indicate standard error of the mean (SEM). (D) Parent (86-028NP) and mutant (RM33) strains were assessed for transformation efficiency in the presence (+) and absence (-) of cAMP as described in Materials and Methods. The transformation efficiency is reported as the percentage of transformed bacteria from the total number of viable bacteria. Sstatistical significance was determined using a two–tailed Student’s *t* test on seven biological replicates. The geometric mean is plotted and error bars represent standard error of the mean (SEM). (E) Parent (86-028NP*rpsL*) and mutant (86-028NP*rpsL icc63A>T*) strains were assessed for transformation efficiency in the absence (-) of cAMP as described in Materials and Methods. Statistical significance was determined using a two–tailed Student’s *t* test on seven biological replicates. The geometric mean is plotted and error bars represent standard error of the mean (SEM).

Icc phosphodiesterase activity was quantified using two different experimental approaches. Purified recombinant proteins (Fig 4A) were incubated with cAMP to quantify the amount of cAMP degraded by Icc and Icc_D21V_ using a modified assay for phosphodiesterase activity [29]. As expected, we observed a statistically significant 200 fold decrease in recombinant Icc_D21V_ activity as compared to the recombinant parental Icc (Fig 4B). Icc is the only cAMP-dependent phosphodiesterase ortholog identified in 86-028NP. Therefore, cytoplasmic extracts from RM33 would be expected to be devoid of any cAMP activity. To this end, cytoplasmic extracts were prepared from exponentially grown cells and the phosphodiesterase activity was quantified as described above. The cytoplasmic extract from RM33 exhibited a statistically significant decrease in phosphodiesterase activity (Fig 4C). The mutations in *icc* that arise during long-term stationary phase culture result in loss of cAMP phosphodiesterase activity and likely increase cAMP levels in the cytoplasm.

### RM33 displays enhanced transformation efficiency

Prior studies demonstrated that cAMP levels regulate competence in *Haemophilus* with increased cAMP resulting in increased transformation efficiency [30, 31]. Our data suggest that the increased cAMP levels in the cytoplasm of RM33 would correlate to an increased rate of uptake of exogenous DNA. Consistent with previous reports, we observed that the addition of exogenous cAMP resulted in a significant increase in the transformation efficiency of the parental strain 86-028NP (Fig 4D). As predicted, in the absence of exogenous cAMP, the transformation efficiency of RM33 was indistinguishable from that of the parent in the presence of exogenous cAMP. To validate these findings, we repeated the assay with a strain in which we genetically reconstructed the *icc* mutation identified in strain RM33. Again, we observed a significant increase in the transformation efficiency of 86-028NP *rpsL icc63A>T* strain in the absence of exogenous cAMP (Fig 4E). While prior studies demonstrate that transformation is increased as cAMP levels are elevated, saturation of cAMP will suppress transformation [30]. As predicted, the addition of exogenous cAMP to RM33 resulted in a decreased transformation efficiency (Fig 4D). Collectively, these data suggest that RM33 exhibits elevated endogenous levels of cAMP due to the mutation in *icc* and the increased ability of RM33 to uptake DNA demonstrates biological significance for the mutation observed in *icc*.

### The absence of effusion is not an indicator of clearance of RM33 from middle ear tissue

**Fig 5:**
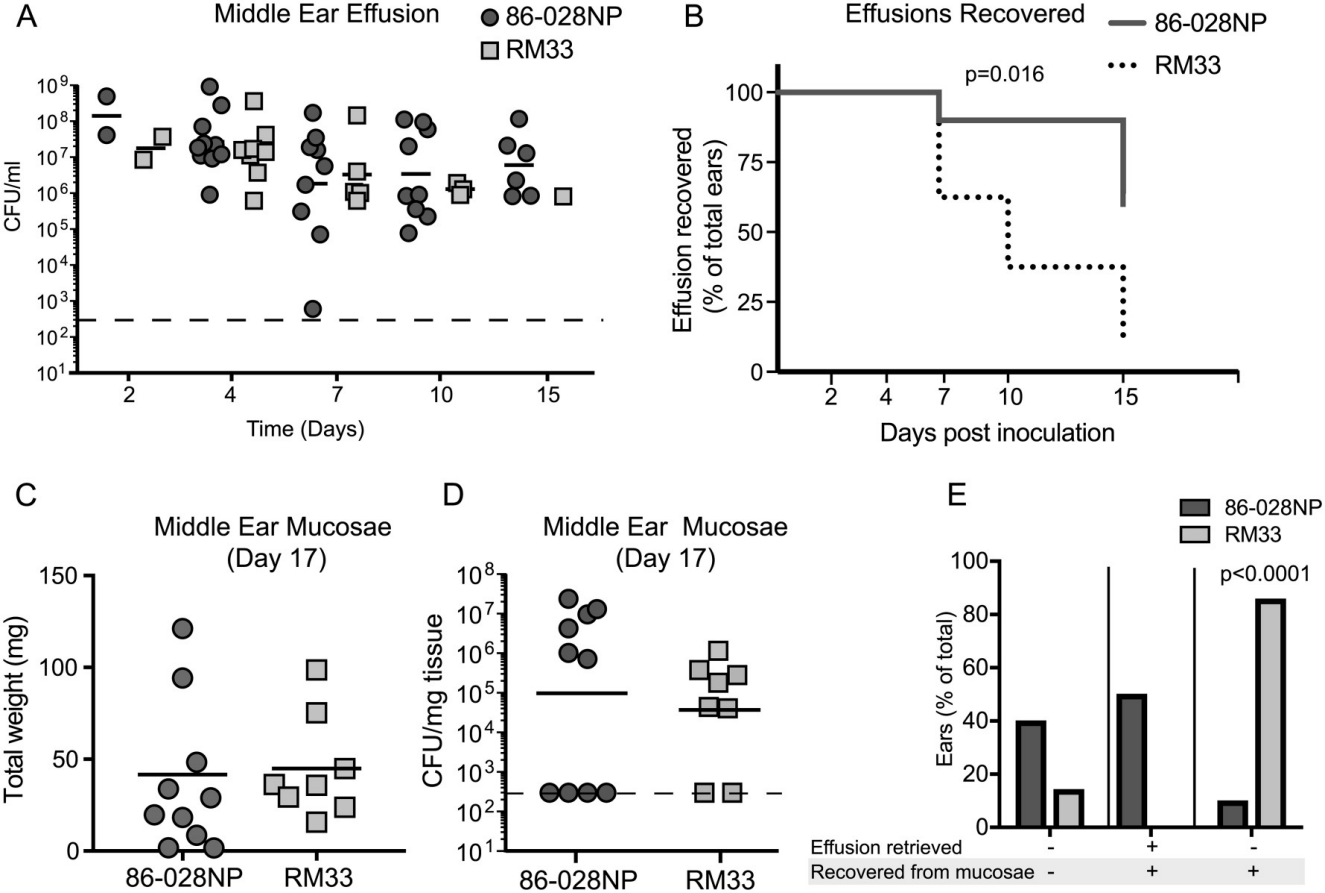
RM33 remains mucosa-associated in the absence of middle ear effusion during experimental OM. (A) Middle ear effusions were retrieved on the days indicated on the x-axis from chinchillas (n = 10 ears for parent, 8 for RM33) experimentally infected with the parent (dark gray circles) or RM33 (light gray squares). Bacterial burden for each effusion was determined by serial dilution and plating and reported as CFU/mL. The absence of data points represents the inability to retrieve middle ear effusion at that time point. There was no statistically significant difference in the bacterial burden for each time point as determined using Mann-Whitney *U* test. Bar represents geometric mean, dashed line indicates the limit of detection. Geometric mean is indicated by the bar. (B) Kaplan Meier curve demonstrating time to clearance of middle ear effusions from the parent cohort (solid line) and RM33 cohort (dotted line). The number of ears with recoverable effusion are reported as percent of the total ears. Statistical significance was determined using a chi square test. (C) The weight of middle ear mucosal tissue including biofilm retrieved from animal cohorts infected with parent (dark gray circles) or RM33 (light gray squares). There was no statistically significant difference in mucosal weight as determined using a Mann-Whitney *U* test. The mean is indicated by the bar. (D) Middle ear muscosal tissues retrieved from animal cohorts infected with parent (dark gray circles) or RM33 (light gray squares) and homogenized to determine bacterial burden reported as CFU/mg tissue. There was no statistically significant difference in bacterial burden as determined using a Mann-Whitney *U* test. (E) The effusion retrieved was correlated with bacteria recovered from middle ear mucosae. Data are reported as the number of cohort ears infected with parent (dark gray bars) or RM33 (light gray bars) as a percentage of the total number of ears in that cohort. Statistical significance was determined with a two tailed binomial test. The geometric mean is indicated by the bar.

Our observations indicate that transient heme-iron restriction promotes survival during long-term stationary phase *in vitro*. We hypothesize that strains adapted to nutrient limitation are more adept for colonization and evasion of host immune responses resulting in persistence. Diagnosis of OM is clinically confirmed by the presence of middle ear fluid (effusion). Clearance of effusion is typically associated with resolution of disease. We initially investigated whether RM33 would alter the disease course as measured by the presence of effusion in the preclinical model of OM. For the first week of infection, we observed no difference in either the presence of effusion or the bacterial burden suggesting that RM33 has similar fitness and kinetics as the parent in the early stages of infection (Fig 5A and 5B). In contrast, 10 days following infection we observed a significant decrease in the number of effusions recovered from animals infected with RM33 (Fig 5B). This decrease is accentuated 15 days following infection when a single effusion was recovered from animals infected with RM33 (Fig 5B). These data suggest that RM33 does not promote chronic disease as determined by the presence of effusion.

The observation that effusion is not recovered from the majority of animals infected with RM33 suggests the bacteria are cleared from the middle ear. To determine the bacterial burden associated with the mucosal tissue and associated extracellular biofilm, we harvested this tissue upon sacrifice (Day 17). Despite the absence of effusion in the ears infected with RM33 (Day 15), we observed similar tissue biomass to that of ears infected with the parent, suggesting that the disease was not resolved (Fig 5C). Homogenates of middle ear mucosae also revealed similar bacterial burdens between the tissues of ears infected with RM33 and the parent (Fig 5D). Clinically, absence of effusion is typically associated with resolution of disease, suggesting the absence of bacteria [32]. As expected for a resolved infection, 40% of ears infected with the parent had no recoverable effusion and no bacteria recovered from middle ear mucosal homogenates (Fig 5E). In contrast, only 14% of ears infected with RM33 exhibited this phenotype (Fig 5E). A recoverable effusion is indicative of an active infection. Effusions could be recovered from 50% of ears infected with the parent, whereas none of the ears infected with RM33 had recoverable effusion. Taken together, 90% of the ears infected with the parent paralleled clinical presentation and resolution of disease, whereas this was observed in only 14% of RM33 infected ears. Remarkably, 86% of ears infected with RM33 had bacteria associated with the middle ear mucosal tissue, yet no effusion was recovered. These observations suggest that the absence of effusion may not be an accurate clinical indicator of resolution of middle ear disease. Furthermore, unlike the parent, RM33 remains associated with the middle ear tissue following resolution of effusion, suggesting that the microevolution of bacteria in response to nutrient limitation may promote subclinical or latent phenotypes.

### NTHI persists in middle ear mucosae as intracellular bacterial communities

**Fig 6:**
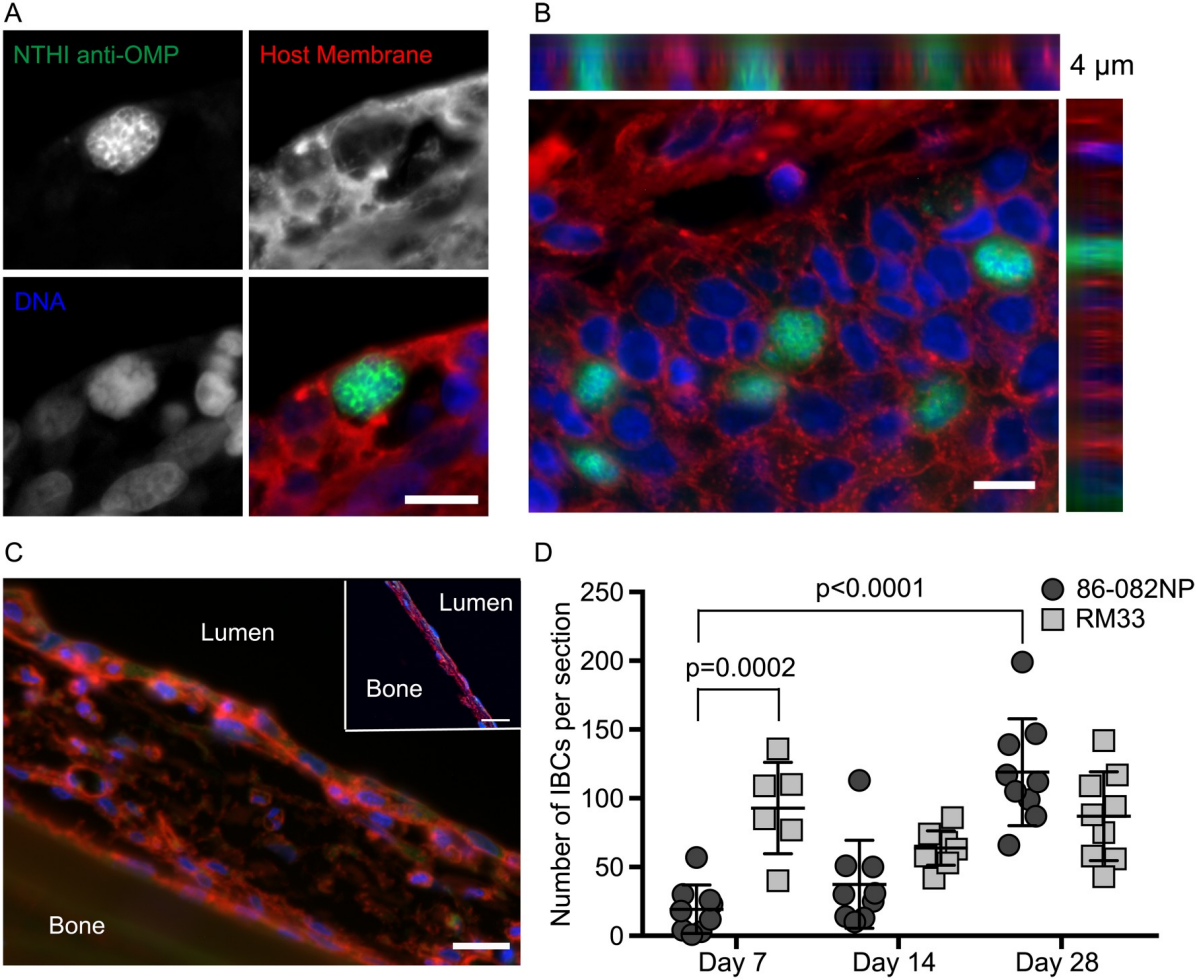
NTHI persists in middle ear mucosae as intracellular bacterial communities (IBCs). Thin sections of middle ears from parent or RM33 cohorts were processed for immunofluorescence microscopy. (A) Immunofluorescence microscopy gallery depicting the surface staining of NTHI by anti-OMP labelling (green), the host cell membrane by wheat germ agglutinin (red), and host and bacterial DNA by Hoescht (blue). Scale bar = 10 μm. (B) 3-dimensional rendering of a series of optical sections to depict the orthogonal views of IBCs formed by RM33 filling the entirety of the epithelial cell and visualized using the fluorophores depicted in panel A. In this image, six IBCs are observed in the 4 micron section. Scale bar = 10 microns. (C) To ensure correct interpretation of observations, infected middle ears were prepared for immunofluorescence microscopy in the absence of antisera directed against NTHI to demonstrate the structures observed were due to the presence of antibody. In addition, sham treated ears were also visualized by immunofluorescence microscopy to ensure that structures were only present in infected ears (inset). (D) The presence of IBCs was enumerated in three thin sections of middle ears infected with the parent (dark gray circles) or RM33 (light gray squares). IBCs were enumerated from three sections from at least three independent animals. Each data point represents the number of IBCs in one thin section. Statistical significance was determined using a Mann-Whitney *U* test. The mean for each data set is indicated and error bars represent standard error of the mean (SEM).

Previous studies demonstrated that NTHI are internalized into epithelial cells and then typically traffic to degradative pathways [11, 33]. Although intracellular bacteria have been observed in biopsy samples of children with OM [34], the potential contribution of viable intracellular NTHI populations to the chronicity and recurrence of OM is unclear. Previously, we showed that transient heme-iron restriction promotes NTHI invasion and formation of IBCs in cultured chinchilla middle ear epithelial cells [7]. Given that RM33 arose from a culture transiently restricted of heme-iron and the association of RM33 with mucosal tissues (Fig 5D and 5E), the potential formation of IBCs in the preclinical model of OM was investigated. IBCs were visualized in thin sections using antisera directed against NTHI outer membrane proteins (green). Counterstains to visualize the host cell membranes (red) and DNA (blue) were also included. We observed communities of NTHI within host cell membranes. The individual bacteria within each community were readily identifiable by the surface staining of the outer membrane proteins outlining and surrounding the diffuse bacterial nucleoid (Fig 6A). Specificity of antibody labeling was determined by staining of sham-treated middle ear tissue and through visualization of middle ear tissues in the absence of primary antibody (Fig 6C). The communities observed filled the entire cell cytoplasm as evidenced by 3D orthogonal views from rendered sequential optical sections (Fig 6B). To determine the kinetics of IBC formation during experimental OM, the number of IBCs within thin sections from ears infected for 7, 14 or 28 days were counted. Numerous IBCs were readily observed in middle ears infected with RM33 as early as Day 7. Although IBCs were observed in middle ears infected with the parent on Day 7, there was a statistically significant increase in the number of IBCs formed by RM33 (Fig 6C). We observed similar numbers of IBCs formed by RM33 throughout the time course evaluated. Interestingly, we observed a significant 6-fold increase in the number of IBCs formed by the parent over the 28 days evaluated. This observation suggests that NTHI adaptation during active OM promotes the formation of IBCs.

### ICC mutations affect phosphodiesterase activity in paired clinical isolates

**Fig 7:**
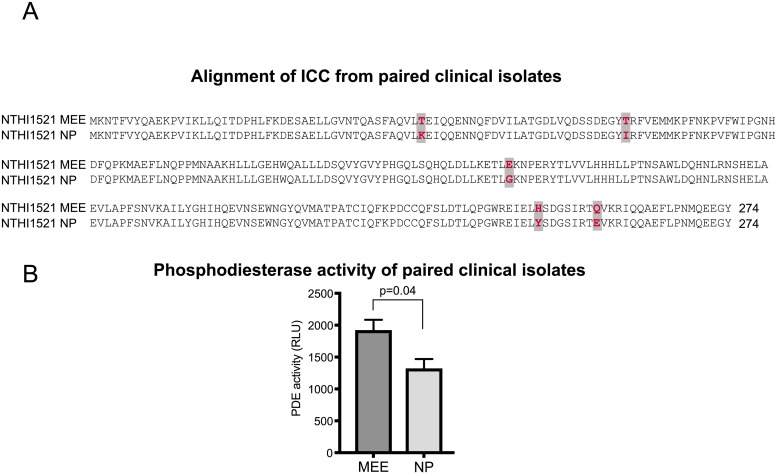
Structural mutations within ICC alter phosphodiesterase activity. (A) Paired NTHI isolates were obtained from middle ear effusion (MEE) or nasopharynx (NP) and the *icc* gene was sequenced as described in the Materials and Methods. Single nucleotide polymorphisms were observed that resulted in 5 amino acid changes (indicated in bold red and gray highlight). (B) Phosphodiesterase activity (PDE) of cytoplasmic extracts was assessed using the PDELight HTS cAMP phosphodiesterase kit and activity reported as relative light units (RLU). Three biological replicates were assayed in technical triplicate and statistical significance was determined using a two–tailed Student’s t test.

To determine whether microevolution of *icc* occurs during disease, we performed an *in silico* comparison of 63 NTHI sequences deposited in Genbank. As expected, we observed regions of Icc that exhibit marked variation in sequence compared with the consensus (S5A Fig), indicating that the *Haemophilus* phosphodiesterase family is heterogeneous in overall protein sequence. We examined the diversity in Icc sequence in NTHI clinical isolate sequences obtained in Genbank and determined that Icc plasticity is greater than that observed in Gyrase, a known housekeeping protein (S5B Fig). These data suggest increased genetic plasticity of proteins under host selective pressures. In contrast to the mutations observed *in vitro* that abolished cAMP phosphodiesterase activity, the amino acids involved in enzyme activity are conserved in all sequences examined, suggesting evolutionary conservation of cAMP phosphodiesterase activity is under selective pressures of the host. It is possible that levels of cAMP phosphodiesterase activity are affected by some of the sequence variations observed which would suggest that adaptation during disease requires modulation of cAMP levels. In order to investigate this, we obtained ten paired NTHI strains that were isolated from the middle ear effusion (MEE) and nasopharynx (NP) of children with active OM. We sequenced *icc* and identified two pairs of strains that contained genetic variations. One of these clinical isolate pairs, NTHI1521MEE and NTHI1521NP, had changes in five amino acids that significantly altered phosphodiesterase actitivity (Fig 7A and 7B). These data indicate that mutations acquired outside the conserved active site of the cAMP phosphodiesterase can modulate enzyme activity and subsequent cAMP concentrations. Further, site specific microenvironments may drive microevolutionary changes in bacterial strains for adaptation at these unique host sites during disease.

## Discussion

The diversity of NTHI lifestyles expands beyond commensalism in the nasopharynx and biofilm formation in the middle ear, to now include intracellular populations of bacteria, a phenotype known to contribute to persistence and recurrence in UPEC, *Proteus*, *Klebsiella* and *Helicobacter* [15]. *In vitro* models have revealed the potential importance of intracellular NTHI, but in these models NTHI typically traffics through the endolysosomal pathway [11, 33, 35]. We have previously demonstrated that transient heme-iron restriction promotes IBC formation in cultured middle ear epithelial cells *in vitro* [7]. Here, we now provide evidence that NTHI completely fills the cytoplasm of middle ear epithelial cells during experimental OM. Thus far, IBCs have primarily been attributed to pathogens of the urinary tract, but our studies now reveal IBCs formed by a pathogen of the respiratory tract. Thus, our studies expand upon previous *in vitro* models and use a preclinical model of OM to provide mechanistic insight into an understudied intracellular niche and the potential contribution to persistence and recurrence.

IBCs formed by UPEC and other species confer tolerance to antibiotics, as well as protection from innate and adaptive immune responses [14, 15]. Further, the intracellular environment provides access to nutrients that are limited in the urine [16, 36, 37]. IBCs are critical for persistence and recurrence for urinary tract infection [15]. These studies have shifted the clinical perspective from a focus on recurrence due to reinoculation to include recurrences from an intracellular bladder reservoir. The implications of these findings have already impacted approaches to treat and prevent recurrent infection [38, 39]. Based upon the UPEC paradigm that IBCs lead to an intrabladder reservoir for reinfection, it is of interest to speculate that IBCs in the middle ear provide a viable population of bacteria to initiate recurrent OM in the absence of reinoculation.

NTHI was recovered from middle ear aspirates from 57% of children with persistent OM at all visits during a longitudinal study [40]. Furthermore, the same strain of NTHI was isolated from the same COPD patient in a longitudinal study, despite periods of negative culture [22]. As such, the observed persistence of NTHI at multiple niches requires new approaches to prevent and treat infection. Persistent bacterial populations can arise stochastically, but also as a result of stressors that include, among others, nutrient limitation [41–45]. The use of heme-iron restriction as a nutritional stressor dramatically increases the longevity of NTHI in stationary phase cultures (Figs 1 and 2). In a preclinical model of OM, the strain adapted though transient heme-iron restriction (RM33) exhibited increased IBC formation as compared with the parent, suggesting that adaptation to fluctuations in nutrient availability promotes an intracellular lifestyle.

Our discovery of IBCs *in vivo* combined with the ability of NTHI to form IBCs in cultured epithelial cells allows investigation of differential trafficking of bacteria either to the lysosome or for productive IBC formation. In our study we focused on nutrient limitation, but the complexity of the host environment suggests that other immune stressors may contribute to NTHI microevolution during disease. Ongoing investigations will delineate specific bacterial factors and mechanisms of invasion that promote IBC formation.

In two independent cultures, we observed microevolution of *icc* which encodes the only cAMP phosphodiesterase in *Haemophilus*. Icc degrades cAMP to modulate the intracellular concentration of this important signaling molecule. DNA import is regulated by cytoplasmic cAMP levels [30] and we and others have shown that defects in cAMP phosphodiesterase increases competence [Fig 4D; [30]]. Bacteria can utilize exogenous DNA for horizontal gene transfer, DNA repair, or as a nutritional source [46, 47]. Use as a nutritional source is a compelling concept and is supported by the observations that DNA is readily available in host niches and only 10–15% of the DNA taken up by NTHI is incorporated intact into the genome, the remainder is degraded [48–51]. The link between nutrition, cAMP levels and competence is supported by the requirement for nutrient limitation as an inducer of competence in *Haemophilus* [52]. Moreover, nutrient availability signals catabolite repression to control levels of cAMP through the enzymatic activities of adenylate cyclase and cAMP phosphodiesterase combined with the cAMP receptor protein (CRP) [53, 54]. In addition to the canonical CRP binding site in *E*. *coli* (CRP-N), *Haemophilus* has an additional regulatory site (CRP-S) that uses Sxy for the regulation of competence [55–58]. Thus, there is overlap between CRP-N and CRP-S directed regulation and a close link between sensing nutritional status and regulation of competence in *H*. *influenzae* [59]. Although we used competence as a tool to determine changes in the levels of cAMP in the adapted strain (RM33), the contribution of competence or any other member of the CRP regulon in the phenotypes observed during infection remain to be elucidated.

These studies provide a new model to delineate the role of cAMP in the development of IBCs. In our study, we observe a decrease in cAMP phosphodiesterase activity following nutrient restriction with an increase in persistence, consistent with the role of cAMP and catabolite repression in nutrient limited conditions. In contrast, other studies provide evidence that a decrease in cAMP levels is associated with increased antibiotic-mediated persister cell formation [60, 61]. Thus, the levels of cAMP appear to modulate persistence through multiple mechanisms based upon the type of stressor encountered. Icc is likely not the only target for adaptation *in vivo*. During disease, fluctuation in nutrients as well as immune stressors may influence adaptation through direct modulation of cAMP levels, indirectly through mutation or regulation of other members of the cAMP regulon, or other as yet unidentified processes.

NTHI was not recovered from the mucosal tissues of some ears (Fig 5D), yet we observed IBCs in all sections evaluated (Fig 6C), suggesting that NTHI within IBCs may not be liberated using standard homogenization techniques or that this population is viable but not culturable following intracellular growth. It should be noted that the IBCs counted represent the number present in a 4μm thin section, which suggests that our quantitation will underestimate the magnitude of intracellular NTHI. In fact, recent studies have demonstrated that enumeration of viable bacteria does not accurately represent the number of bacteria within IBCs [62]. Recent studies have also determined that UPEC IBCs are metabolically active in the bladder [63]. Future studies will evaluate the metabolic activity of NTHI in IBCs in the middle ear.

Collectively, our observations reveal microevolution in response to nutrient limitation leading to persistence in long-term culture, and that this adapted strain exhibited a predilection for productive growth within the epithelial environment which resulted in increased IBC formation during experimental OM. Perhaps even more interesting, we observed an increase in IBC formation of the parent at 28 days during experimental OM, suggesting that genotypic or phenotypic adaptation occurs during infection. As the availability of nutrients change due to the dynamic immune response, we speculate that the intracellular environment provides nutrients leading to bacterial proliferation and IBC formation. Moreover, the presence of IBCs during OM provides the first opportunity to evaluate the role of intracellular populations in chronicity and quiescence as a new paradigm for recurrent OM. This model provides a new platform for the identification of novel diagnostic and therapeutic approaches for OM. Our prior proteomic studies demonstrate an increase in the actin filament branching protein, Arp2/3, during acute experimental OM and that inhibition of Arp2/3 decreases NTHI invasion as a potential adjunct therapy [64]. Finally, it is of interest to determine whether intracellular populations contribute to the other NTHI-mediated diseases such as conjunctivitis, sinusitis, pneumonia, and exacerbations in COPD and cystic fibrosis [65, 66].

## Materials and methods

### Strains and media

NTHI strain 86-028NP is a minimally passaged clinical isolate which has been sequenced and extensively characterized in the chinchilla model of human otitis media [12, 67–70]. NTHI strain RM33 was obtained via heme-iron restriction and isolated on day 33 of long-term culture of NTHI strain 86-028NP. DIS is an RPMI 1640-based medium that was further modified by treatment with 6% Chelex 100 (Sigma, St.Louis, MO) overnight with stirring to remove divalent cations, adjusted to a pH of 7.4 and filtered through a 0.22 μm GPExpress PLUS membrane filter (Millipore, Billerica, MA) and then supplemented with 0.07 mM CaCl_2_, 0.7 mM MgSO_4_ and 2 mg haemin chloride mL^-1^(Sigma Aldrich, St. Louis, MO), generating a DIS medium.

The 86-028NP*rpsL icc*63A>T mutant strain was generated using a recombineering method developed for *Haemophilus* [71]. The *icc* gene in 86-028NP*rpsL* was deleted and replaced by a cassette containing a spectinomycin resistance gene and a copy of *rpsL* from *Neisseria gonorrhoeae* (*rpsL*_*Ng*_). The *icc* gene including approximately 1kb of DNA flanking sequences were cloned into pGEM-T Easy (Promega, Madison, WI). The *icc*63A>T mutation was introduced into *icc* using QuikChange (Agilent, Santa Clara, CA). This construct was linearized by SapI digestion and introduced into 86-028NP*rpsL* Δ*icc*::spec-*rpsL*_*Ng*_ using the MIV method [72]. After transformation, clones containing chromosomal *icc63A>T* were selected on chocolate agar supplemented with 1000 μg/mL streptomycin. Loss of the cassette was confirmed by screening clones on chocolate plates containing 200 μg/mL spectinomycin and the presence of *icc*63A>T was confirmed by sequencing. A complete list of strains and primers used in this study is provided (S1 and S2 Tables).

Our approach was to initially determine the role of *icc* during experimental OM. To this end, parallel cohorts of chinchillas received either a single infection or a co-infection of the parent and RM33. To distinguish the parent from RM33 in the competition model, we engineered 86-028NP and RM33 to carry antibiotic resistance markers as previously described [73]. From this first series of studies, we observed differences in host responses in the ears infected with both strains as compared with the ears infected with either of the strains independently, suggesting that there were dominant phenotypes that resulted in premature clearance of the bacteria in the co-infection model. Therefore only single infections were used to determine the kinetics of infection of RM33, with either marked or unmarked strains.

NTHI1521 MEE and NTHI1521 NP were kindly provided by Dr. Lauren Bakeletz. These strains were obtained from the middle ear effusion (MEE) and the nasopharynx (NP) from the same child with active otitis media. Using multi-locus sequence testing and outer membrane protein profiles, we determined that these two strains represent different sequence types. NTHI1521MEE was determined to be ST329 and a member of clonal complex 3 while NTHI1521NP was ST165 and a member of clonal complex 3. Sequence analysis of *icc* revealed that there were 5 amino acid changes, none of which were in the active site, but still appear to alter cAMP phosphodiesterase activity.

For routine culturing on agar plates, strains were grown on chocolate agar (Becton Dickinson, Sparks, MD). Marked strains were grown on chocolate agar plates supplemented with 200 μg/mL of spectinomycin or 20 μg/mL of kanamycin. All growth was at 37°C in 5% CO_2_, unless stated otherwise. For routine liquid culture, cells were grown in BHI supplemented with 2 μg/mL of NAD and 2 μg/mL of heme (sBHI). For persistence assays bacteria were grown in defined iron source (DIS) medium in the presence or absence of 2 μg/mL heme [74] as indicated in the text.

For expression of recombinant Icc, *Escherichia coli* BL21 (DE3) cells (Thermo Fisher Scientific) containing plasmid vectors were grown in L broth supplemented with 50 μg/mL ampicillin.

### MLST

Strain types (STs) for NTHI1521MEE and NTHI1521NP were determined by multilocus sequence typing. Briefly, seven housekeeping genes (adkI, atpG, frdB, fucK, mdh, pgi and recA) were PCR amplified from genomic DNA and sequenced. Allele numbers for each gene and an ST for each strain was generated using the databases at https://pubmlst.org/hinfluenzae/. The clonal complexes that NTHI1521MEE and NTHI1521NP are members are were determined using eBURST (eburst.mlst.net).

### Environmental heme-iron restriction and long-term survival

Environmental heme-iron restriction was performed as previously described [7]. Briefly, 86-028NP was grown overnight on chocolate agar (Becton Dickinson, Sparks, MD). Individual colonies were suspended in chelated defined iron source (DIS) medium, prepared as previously described [70], to an OD_490_ of 0.65, then diluted 10-fold into 15 mL round bottom glass tubes (nitric acid-washed to remove all metals) containing DIS medium with either 0, or 2 μg/mL heme (Millipore Sigma, Billerica, MA). Following 24 hours incubation, cultures were adjusted to an OD_490_ of 0.37 in DIS medium and diluted 10-fold into 5 mL DIS medium containing 2 μg/mL heme. For this study, to evaluate long-term persistence, cultures were incubated statically days with no fresh medium added. Every 24 hours, cultures were vortexed at 300 rpm for 2 seconds. Cultures were serially diluted and plated on chocolate agar to assess viability. The individual culture was terminated when the culture medium was depleted or no bacterial colonies were observed after at least three consecutive days of plating. Each day, bacteria were collected from plates and cryopreserved by resuspending in 1 mL 20% glycerol, 0.8% skim milk and stored at -80°C to generate a series of isolates (RM series). RM33 (isolate collected on day 33) was used in subsequent experiments. A second, parallel and independent long-term survival assay was completed using the same methodology and a second series of isolates (AR Series) was collected.

### Whole genome sequencing of long-term survival isolates

At each time point the colonies from the enumeration plates were pooled and frozen for long term storage. To purify genomic DNA, a loop full of cells from the frozen stock was grown on chocolate agar overnight. The resultant conlonies were then pooled, lysed and genomic DNA was purified using a Gentra Puregene Yeast/Bact. Kit (QIAGEN, Germantown, MD). Sequencing libraries were prepared and sequenced using paired end 300bp chemistry on the MiSeq platform to high coverage on a single flow cell (Illumina, San Diego, CA). Nucleotide variants were identified using the Churchill algorithm [75] and the percentage of discordant reads as compared to the 86-028NP reference genome was calculated. Single nucleotide variants were independently validated by Sanger sequencing. *icc* was amplified by PCR using the following primers AH0422 and AH0423 (S1 Table). The amplicons were then purified using a QIAquick PCR Purification Kit (QIAGEN) and sequenced by Eurofins Genomics (Louisville KY), using the amplification primers as sequencing primers. Sequence data was assembled using SeqMan Pro (DNASTAR, Madison, WI) and nucleotide variants identified through comparison with the parental gene sequence.

### Tertiary structure prediction and validation of 3',5'-cyclic adenosine monophosphate phosphodiesterase Icc

SWISS- MODEL was used to model the 3',5'-cAMP phosphodiesterase Icc from 86-028NP using the automated modeling mode [76]. Templates were populated based on a search for evolutionarily related structures matching the target sequence, with the model built based on the target-template alignment using ProMod3 Version 1.1.0 and quality assessed. The quality of the predicted model was further evaluated using the structure assessment program SAVES (https://services.mbi.ucla.edu/SAVES/), PROCHECK [77], ERRAT [78], VERIFY_3D [79], PROVE [80], and Ramachandran Plot. Computation models were prepared and rendered with the PyMOL Molecular Graphics System (DeLano Scientific, San Carlos, CA, 2002).

### Purification of recombinant Icc

The coding sequence of *icc* from 86-028NP was PCR amplified with a primer that introduced an NdeI restriction site at the 5’ end of the gene (AH0440, S1 Table) and a second primer that introduced the BamHI restriction site at the 3’ end of the gene (AH0441, S1 Table). The latter primer also removed the stop codon to produce Icc with a C-terminal His-tag. Using NdeI and BamHI, the amplicon was cloned as a first codon fusion in pET15b (Millipore Sigma) and transformed into *E*. *coli* BL21 (DE3) cells (Thermo Fisher Scientific). The point mutation at nucleotide 63 in *icc* from RM33 was introduced into pET15b-*icc* by QuikChange. This construct was also transformed into *E*. *coli* BL21 (DE3). To express and overproduce recombinant Icc (rIcc), bacteria were grown in L broth at 37°C with shaking at 200 rpm. When bacteria were in mid-exponential phase, IPTG (Thermo Fisher Scientific) was added to a final concentration of 1mM and bacteria were incubated at 37°C with shaking at 200 rpm for an additional four hours. Bacteria were then harvested by centrifugation at 6,000xg for 15 minutes, resuspended in lysis buffer (50 mM NaH_2_PO_4_, 300 mM NaCl, pH 8.0) and lysed by one passage through a high-pressure cell (25,000 psi; One Shot Model, Constant Systems Ltd., Kennesaw, GA). Insoluble material was removed by centrifugation at 16,000xg for 20 minutes and rICC purified on High Affinity Ni-Charged Resin following the manufacturer’s protocol (GenScript, Piscataway, NJ). Protein concentration was assessed using Coomassie Plus (Bradford) Assay Reagent (Thermo Fisher Scientific) then resolved on a 4–20% Mini-PROTEAN TGX Precast Gel (Bio-Rad, Hercules, CA) and silver stained (Pierce Silver Stain Kit, Thermo Fisher Scientific) to assess protein purity.

### Isolation of soluble proteins

Bacteria were grown in sBHI at 37°C with shaking at 180 rpm. When bacterial cells were in mid-exponential phase, they were harvested by centrifugation at 6,000xg for 15 minutes then resuspended in 10 mM HEPES, pH 7.4 and lysed by one passage through a high-pressure cell (25,000 psi; One Shot Model, Constant Systems Ltd). Insoluble material was removed by centrifugation at 16,000xg for 20 minutes, the clarified supernatant collected and protein concentration assessed using Coomassie Plus (Bradford) Assay Reagent (Thermo Fisher Scientific).

### Analysis of Icc function by phosphodiesterase activity

To measure cAMP dependent phosphodiesterase activity, purified parent and mutant recombinant Icc proteins were buffer exchanged and concentrated into 50mM Tris pH 7.6, 0.1mM DTT, 10 μM FeCl_3_ [29] using an Amicon Ultra 4 10K Ultracel Centrifugal Filter (Millipore Sigma) after which protein concentration was assessed using Coomassie Plus (Bradford) Assay Reagent. cAMP phosphodiesterase activity was measured using a PDELight HTS cAMP phosphodiesterase Kit (Lonza, Morristown, NJ). Thirty nine microliters of 10 μM cAMP was mixed with 2 ng of recombinant Icc, to give a final volume of 40 μL. Samples were incubated for 30 minutes at room temperature then 20 μL of AMP detection reagent was added. After a further incubation for 10 minutes at room temperature, luminescence was quantified using a Synergy Hybrid H1 Reader (BioTek, Winooski, VT; default luminescence setting with a 0.1 second integration time). Three biological replicates were assessed in technical triplicate and statistical significance was determined using a one–tailed Student’s *t* test (Graph Pad Prism, La Jolla, CA).

### Analysis of phosphodiesterase activity in cell lysates

cAMP-dependent phosphodiesterase activity was quantified in lysates from 86-028NP and RM33 that contained both cytoplasmic and periplasmic proteins. Using the protocol outlined for rIcc, the activity in 100 ng of lysate was assessed. Four biological replicates were tested in technical triplicate and statistical significance was determined using a one–tailed Student’s *t* test (Graph Pad Prism).

### Transformation efficiency experiments

To assess the transformation efficiency of the parent and RM33 isolates, we used recombineering [71] to generate a mutation in a putative sRNA–encoding gene, sRNA121, located in the intergenic region between NTHI0351 and NTHI0353. sRNA 121 and approximately 1kb of flanking DNA was cloned into pGEM-T Easy. sRNA121 was then deleted and replaced by a cassette comprising a spectinomycin resistance gene and an *rpsL*_*Ng*_ gene. This construct, when linearized by NcoI digestion, was used to determine strain transformability using a modified MIV method in the presence or absence of cAMP [72]. After transformation, bacteria were grown on chocolate agar to enumerate the total viable population, as well as on chocolate agar that contained 200 μg/mL spectinomycin, to enumerate the transformed population. The transformation efficiency is reported as the percentage of transformed bacteria from the total number of viable bacteria. Seven biological replicates were assayed and statistical significance was determined using a two–tailed Student’s *t* test (Graph Pad Prism, La Jolla, CA).

### Ethics statement

Animal experiments were completed in adherence to the accredited conditions in the Guide for the Care and Use of Laboratory Animals of the National Institutes of Health. The protocol was approved by the Institutional Animal Care and Use Committee at the Research Institute at Nationwide Children’s Hospital (Welfare Assurance Number A3544-01), AR13-00026. All experimental procedures were performed under anesthesia (xylazine and ketamine administration) and all efforts made to minimize suffering.

### Preclinical models of otitis media

#### Time course to determine kinetics of single strain infections

NTHI strains 86-028NP(pSPEC1) and RM33(pGZRS-39A) were grown on chocolate agar overnight. NTHI were resuspended in 0.9% (w/v) sodium chloride in non-pyrogenic sterile water (Pfizer, New York NY) to an optical density of 0.65 measured at 490 nm and diluted for inoculation. Two cohorts of five chinchillas each were transbullarly inoculated in each ear with 300 μL containing either 934 colony forming units (CFUs) of 86-028NP(pSPEC1) or 1070 CFUs of RM33(pGZRS-39A). On days 7, 10, and 15 post-inoculation, middle ear effusions were collected by epitympanic tap from the inferior bullae. Middle ear effusions were serially diluted and plated on chocolate agar as well as on chocolate agar supplemented with 200 μg/mL spectinomycin or 20 μg/mL kanamycin. On day 17 post-inoculation, chinchillas were sacrificed and the middle ear mucosal tissues were removed, weighed, homogenized and serially diluted and plated on antibiotic-supplemented chocolate agar plates. Viable CFUs from middle ear effusions and tissue homogenates were counted to determine burden of infection. The bacterial burden of tissue homogenates was normalized for total tissue weight.

#### Visualization of intracellular bacterial communities in middle ear mucosal tissue

To quantitate the formation of IBCs as a result of infection with 86-028NP or RM33, two cohorts of 9 chinchillas each were transbullarly inoculated with 640 CFUs of 86-028NP or 386 CFUs RM33. On days 7, 14, and 28, chinchillas were sacrificed and the middle ears removed, fixed in 4% paraformaldehyde in Dulbecco’s Phosphate Buffered Saline (DPBS), decalcified in 0.35 M Tris/EDTA solution and embedded in paraffin. Thin sections from fixed middle ears were deparaffinized in xylene and antigen-retrieval was performed as previously described [64] followed by treatment with 0.01% sodium borohydride (Thermo Fisher Scientific) in DPBS for 10 minutes to inhibit inherent fluorescence due to aldehyde production. Slides were then incubated for 10 minutes in CAS Block (Thermo Fisher Scientific) to prevent non-specific staining followed by a 30-minute incubation with Image-iT FX Signal Enhancer (Thermo Fisher Scientific). NTHI were labeled with a chinchilla primary antibody raised against total outer membrane proteins [OMP; [12]], diluted 1:25 in CAS Block overnight at 4°C. NTHI was visualized using Protein A conjugated with Alexa Fluor 488 (Thermo Fisher Scientific) diluted 1:100 in CAS Block and incubated at room temperature for one hour. Host cell membranes were visualized with wheat germ agglutinin conjugated with Alexa Fluor 594 (Thermo Fisher Scientific) diluted 1:200 in DPBS. DNA was counterstained with Hoescht 33342 (Thermo Fisher Scientific) diluted 1:300 in DPBS. Photobleaching was reduced by the addition of ProLong Gold Antifade (Thermo Fisher Scientific) prior to addition of the coverslip and tissues were imaged with an Axiovert 200M inverted epifluorescence microscope equipped with the Apotome attachment for improved fluorescence resolution (Carl Zeiss, Inc., Thornwood, NY). IBCs were quantified by manually counting the total number of infected cells in each of three thin sections for each cohort. Statistics were performed using a two-tailed Student’s *t* test (GraphPad Prism, La Jolla, CA).

## Supporting information

S1 Fig (A). Alignment of representative Sanger sequences of *icc* from the continuously exposed cultures of the RM series. (B) Alignment of the Sanger sequences of *icc* from a third independent persister experiment demonstrating the introduction of a stop codon in the culture that originated from the transiently restricted culture (TR) and the parental sequence of the culture that originated from the contiuosly exposued (CE) culture on day 14.
(TIF)

S2 Fig Individual strains indicated were grown overnight in the presence (CE) or absence (TR) of 2 μg/mL heme-iron and diluted to OD_490_ at 0.05 in fresh medium containing 2 μg/mL heme-iron for growth at 37 °C under static conditions. Every hour, samples were removed for serial dilution and plating to enumerate the number of viable bacteria (n = 2) (A) or turbidity of the culture was determined as the optical density at 490 nm (B). A representative of two independent experiments is depicted.
(TIF)

S3 Fig Individual strains indicated were grown overnight in the presence (CE) or absence (TR) of 2 μg/mL heme-iron and diluted to OD_490_ at 0.05 into fresh medium containing 2 μg/mL heme-iron. Equal volumes of the indicated cultures were then mixed for growth at 37 °C under static conditions Every hour, samples were removed for serial dilution and plating to enumerate the number of viable bacteria (n = 2)(A-C) or turbidity of the culture was determined as the optical density at 490 nm (D). A representative of two independent experiments is depicted.
(TIF)

S4 Fig Strains indicated were grown overnight in the presence (CE) or absence (TR) of heme-iron and diluted to OD_490_ at 0.05 in fresh medium for growth at 37 °C under static conditions. Every day, samples were removed for serial dilution and plating to enumerate the number of viable bacteria. (A) Assessment of long term viability of individual cultures. (B-D) Evaluation of co-culture of strains indicated.
(TIF)

S5 Fig (A) Sequences of *icc* from NTHI were obtained from published sequences in GenBank and aligned using CLUSTAL W. The consensus sequence was determined for all sequences. Residues identical to the majority consensus are shaded in black. Residues that do not match the majority consensus are indicated in white. The NTHI strains for which the genome is complete are highlighted in red. (B) Sequences of Icc and GyrA from NTHI were obtained from published sequences in GenBank.
(PDF)

S1 Table List of strains obtained or generated in this study. (EPS)

S2 Table List of primers used in this study. (EPS)

S1 Methods Methodology for growth curve and long-term survival experiments when cultured alone or in co-culture. (DOCX)
