# Supplementary figures and images for: Microevolution in response to transient heme-iron restriction enhances intracellular bacterial community development and persistence
Source: PLoS Pathog. 2018 Oct 17;14(10):e1007355. doi: 10.1371/journal.ppat.1007355 (PMC6205647; doi:10.1371/journal.ppat.1007355)

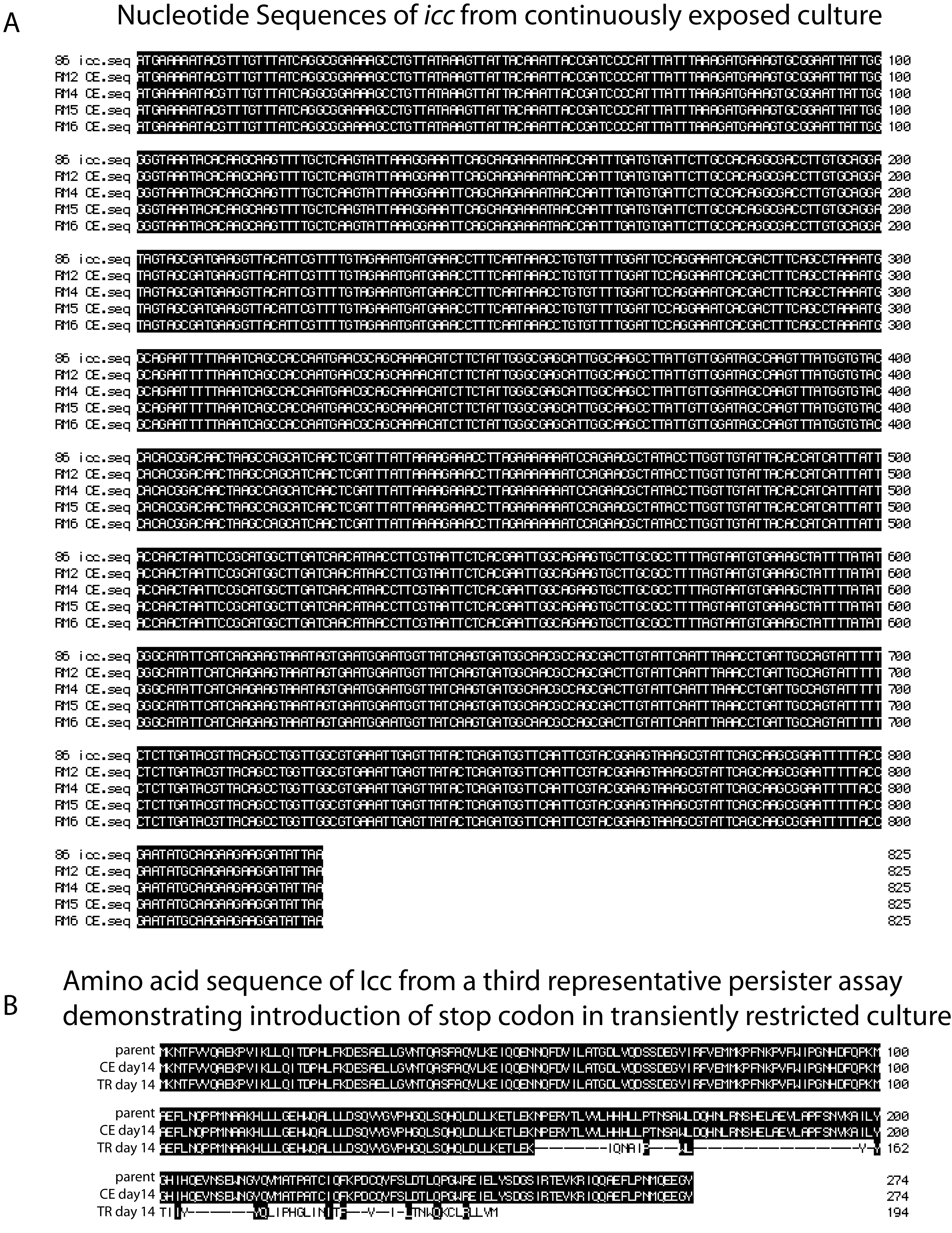

Supplement: S1 Fig — (A). Alignment of representative Sanger sequences of icc from the continuously exposed cultures of the RM series. (B) Alignment of the Sanger sequences of icc from a third independent persister experiment demonstrating the introduction of a stop codon in the culture that originated from the transiently restricted culture (TR) and the parental sequence of the culture that originated from the contiuosly exposued (CE) culture on day 14. (TIF) [file ppat.1007355.s001.tif]

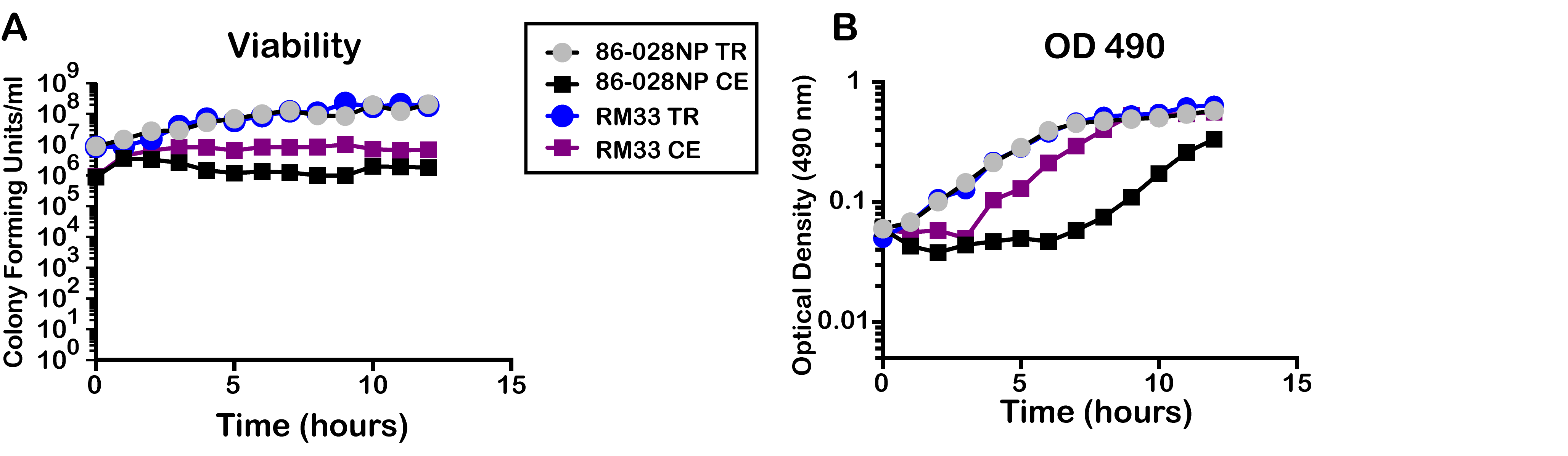

Supplement: S2 Fig — Every hour, samples were removed for serial dilution and plating to enumerate the number of viable bacteria (n = 2) (A) or turbidity of the culture was determined as the optical density at 490 nm (B). A representative of two independent experiments is depicted. (TIF) [file ppat.1007355.s002.tif]

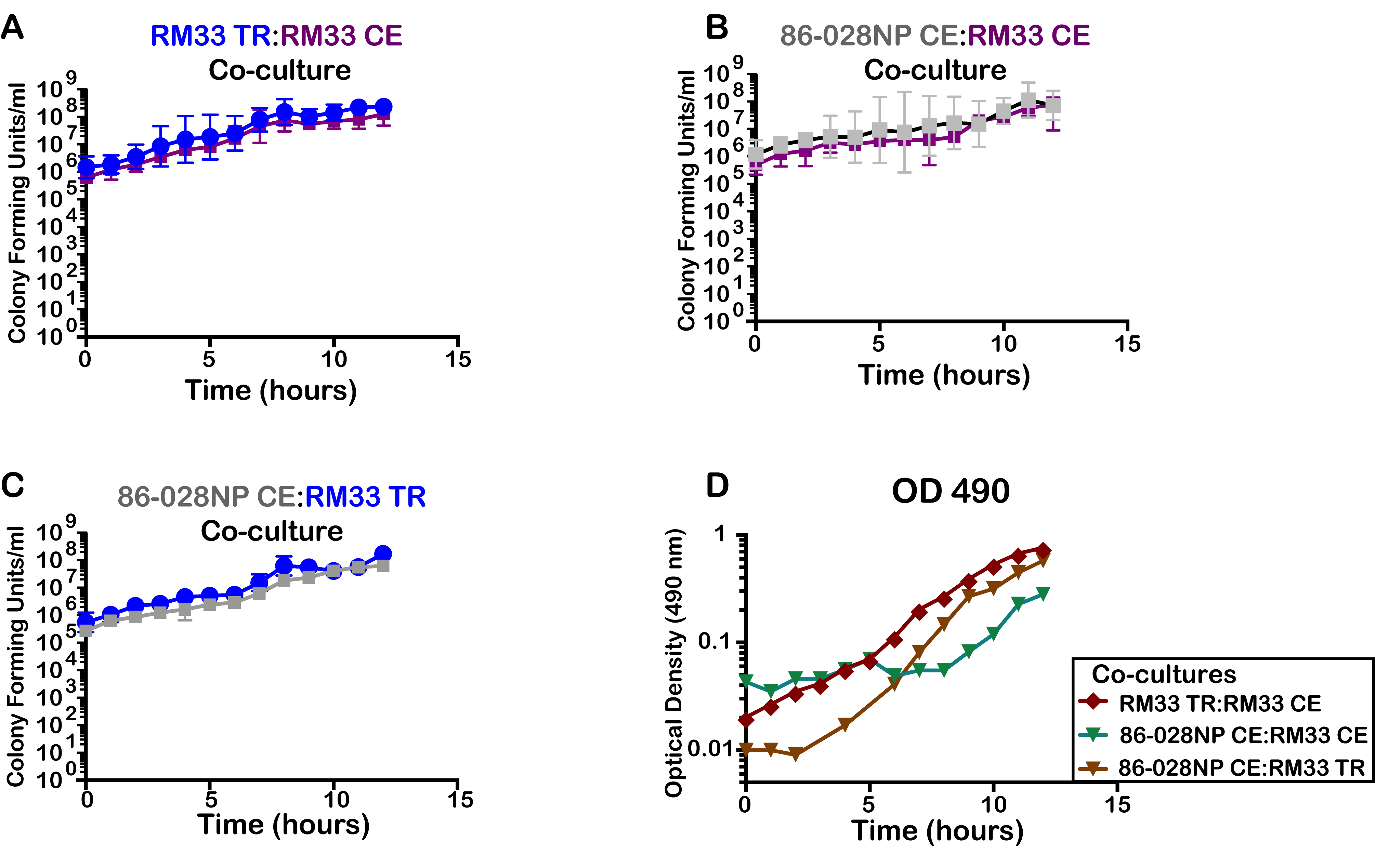

Supplement: S3 Fig — Equal volumes of the indicated cultures were then mixed for growth at 37 °C under static conditions Every hour, samples were removed for serial dilution and plating to enumerate the number of viable bacteria (n = 2)(A-C) or turbidity of the culture was determined as the optical density at 490 nm (D). A representative of two independent experiments is depicted. (TIF) [file ppat.1007355.s003.tif]

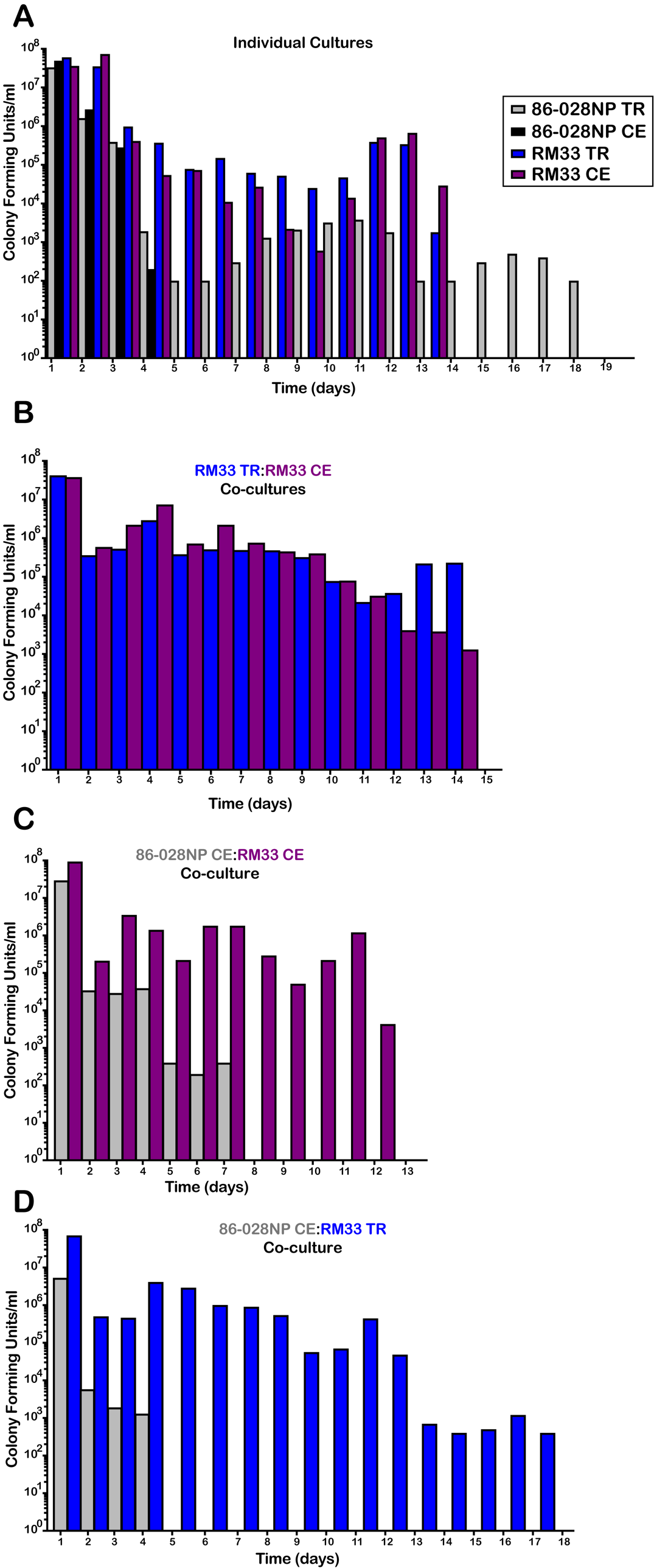

Supplement: S4 Fig — Every day, samples were removed for serial dilution and plating to enumerate the number of viable bacteria. (A) Assessment of long term viability of individual cultures. (B-D) Evaluation of co-culture of strains indicated. (TIF) [file ppat.1007355.s004.tif]

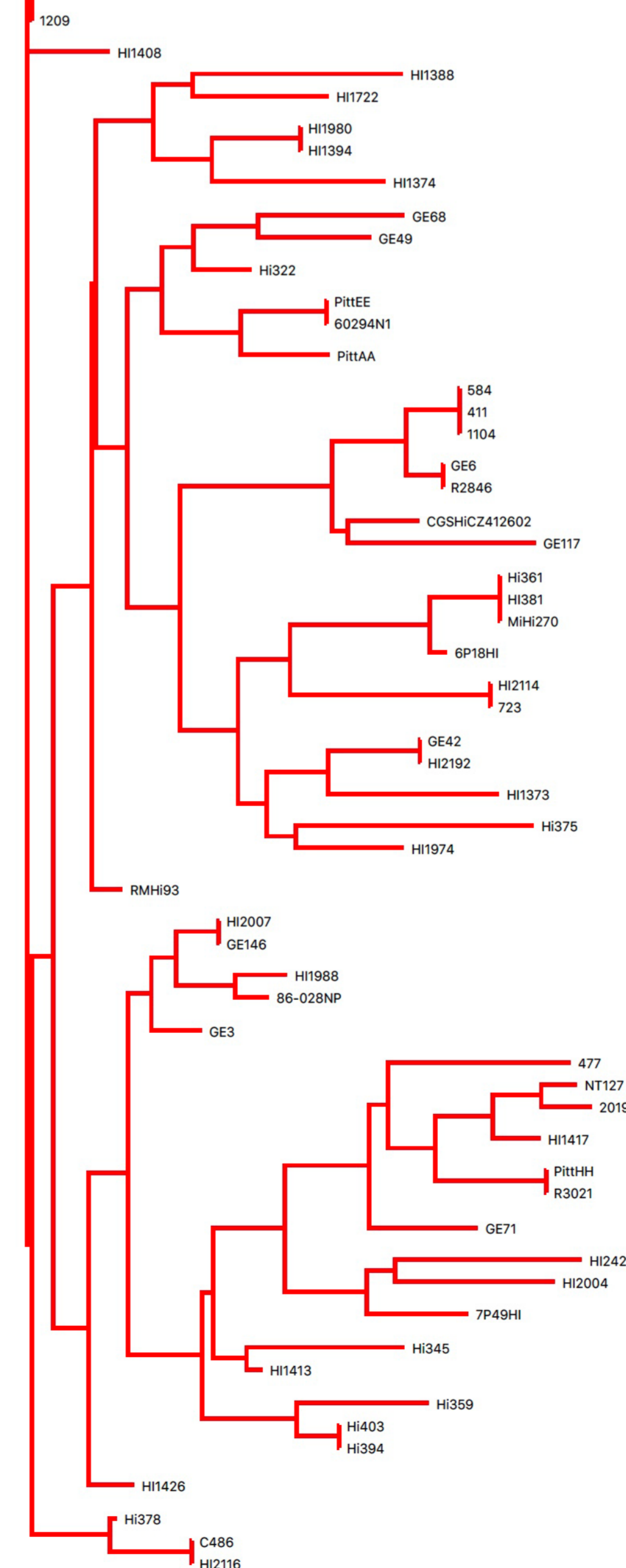

Supplement: S5 Fig — (A) Sequences of icc from NTHI were obtained from published sequences in GenBank and aligned using CLUSTAL W. The consensus sequence was determined for all sequences. Residues identical to the majority consensus are shaded in black. Residues that do not match the majority consensus are indicated in white. The NTHI strains for which the genome is complete are highlighted in red. (B) Sequences of Icc and GyrA from NTHI were obtained from published sequences in GenBank. (PDF) [file ppat.1007355.s005.pdf]
