# Supplementary material for: Microevolution in response to transient heme-iron restriction enhances intracellular bacterial community development and persistence
Source: PLoS Pathog. 2018 Oct 17;14(10):e1007355. doi: 10.1371/journal.ppat.1007355 (PMC6205647; doi:10.1371/journal.ppat.1007355)
Supplement: S1 Methods — (DOCX) [file ppat.1007355.s008.docx]

**Supplemental Methods:**

***Growth curves of nutritionally conditioned persistent NTHI individual and co-cultures***

Environmental heme-iron restriction was performed as previously described (7). Briefly, NTHI strains 86-028NP and RM33 were grown overnight on chocolate agar (Becton Dickinson, Sparks, MD). Individual colonies were suspended in chelated DIS medium to an OD_490_ of 0.65, then diluted 10-fold into 15 mL round bottom glass tubes (nitric acid-washed to remove all metals) containing DIS medium with either 0, or 2 µg/mL heme (Millipore Sigma, Billerica, MA). Following 24 hours incubation, cultures were adjusted to an OD_490_ of 0.05 in 5 ml DIS medium containing 2 µg/mL heme for growth at 37°C under static conditions. To evaluate the role of co-infection on growth kinetics, co-cultures were established at a 1:1 ratio. To distinguish the two strains in the co-culture model, we engineered 86-028NP and RM33 to carry antibiotic resistance markers as previously described (73). Both 86-028NP and RM33 were transformed with pGZRS-39A, a *Haemophilus-Actinobacillus pleuropneumoniae* shuttle vector that contains a kanamycin resistance gene; or pSPEC1, a variant of pGZRS-39A in which the kanamycin resistance gene was replaced by a spectinomycin resistance gene (73). For both individual and co-cultures, growth and viability was determined over a 12-hour period by reading absorbance at 490nm and serially diluting and plating in triplicate on chocolate agar plates containing either 0 ug/mL antibiotic, 20 ug/mL kanamycin or 200 ug/mL spectinomycin.

***Long-term survival of nutritionally conditioned persistent NTHI individual and co-cultures***

In parallel to experiments evaluating the growth and viability of individual and co-cultures of persistent NTHI over 12 hours, additional cultures were prepared to evaluate the long-term survival of the cultures. Transiently restricted or continuously exposed NTHI cultures were adjusted to an OD_490_ of 0.05 in 5 ml DIS medium containing 2 µg/mL heme for growth at 37°C under static conditions. Co-cultures were established as described for growth curve experiments using genetically engineered strains carrying antibiotic resistance markers and prepared in a 1:1 ratio. Every 24 hours, viability was determined by serially diluting and plating in triplicate on chocolate agar plates with or without antibiotic for selection.
